# Innate Immune Determinants of Graft-Versus-Host Disease and Bidirectional Immune Tolerance in Allogeneic Transplantation

**DOI:** 10.21926/obm.transplant.1901044

**Published:** 2019-01-31

**Authors:** Anouk A. J. Hamers, Sunil K. Joshi, Asha B. Pillai

**Affiliations:** 1. Department of Pediatrics, Division of Hematology / Oncology and Bone Marrow Transplantation, University of Miami Miller School of Medicine, Miami, FL, USA;; 2. Batchelor Children’s Research Institute, University of Miami Miller School of Medicine, Miami, FL, USA;; 3. Department of Microbiology & Immunology, University of Miami Miller School of Medicine, Miami, FL, USA;; 4. Sylvester Comprehensive Cancer Center, University of Miami Miller School of Medicine, Miami, FL, USA;; 5. Holtz Children’s Hospital, University of Miami Miller School of Medicine, Miami, FL, USA.

**Keywords:** Innate immune cells, central tolerance, peripheral tolerance, allo-transplantation, Graft-versus-Host Disease, rejection, thymic selection, regulatory T Cells, immunosuppression, mixed chimerism

## Abstract

The success of tissue transplantation from a healthy donor to a diseased individual (allo-transplantation) is regulated by the immune systems of both donor and recipient. Developing a state of specific non-reactivity between donor and recipient, while maintaining the salutary effects of immune function in the recipient, is called “immune (transplantation) tolerance”. In the classic early post-transplant period, minimizing bidirectional donor ←→ recipient reactivity requires the administration of immunosuppressive drugs, which have deleterious side effects (severe immunodeficiency, opportunistic infections, and neoplasia, in addition to drug-specific reactions and organ toxicities). Inducing immune tolerance directly through donor and recipient immune cells, particularly via subsets of immune regulatory cells, has helped to significantly reduce side effects associated with multiple immunosuppressive drugs after allo-transplantation. The innate and adaptive arms of the immune system are both implicated in inducing immune tolerance. In the present article, we will review innate immune subset manipulations and their potential applications in hematopoietic stem cell transplantation (HSCT) to cure malignant and non-malignant hematological disorders by inducing long-lasting donor ←→ recipient (bidirectional) immune tolerance and reduced graft-versus-host disease (GVHD). These innate immunotherapeutic strategies to promote long-term immune allo-transplant tolerance include myeloid-derived suppressor cells (MDSCs), regulatory macrophages, tolerogenic dendritic cells (tDCs), Natural Killer (NK) cells, invariant Natural Killer T (iNKT) cells, gamma delta T (γδ-T) cells and mesenchymal stromal cells (MSCs).

## 1. Introduction

Clinical allograft rejection was first reported by Earl C. Padgett in 1932, when he applied unrelated skin grafts to treat burn patients. Padgett noted that, whereas skin grafts derived from donors unrelated to the recipients were rejected within a short time, grafts deriving from close relatives were not immediately rejected by the transplant recipient. This was the first observation of an effect of genetic compatibility between donor and recipient on graft survival, a phenomenon mechanistically explained when the immunological basis of allo-rejection was later elucidated by Gibson and Medawar in 1943 [1–3]. In 1948, Medawar and colleagues made the key observation that cellular components of the recipient immune system are responsible for the rejection of the graft and that this phenomenon was subject to some form of immunologic “memory” [4–7]. Medawar’s observation was complemented in 1949 by Burnet’s theory that the immune system can discriminate between foreign antigens (i.e. “non-self” antigens) and antigens derived from one’s own body (i.e. “self” antigens) [1, 3]. In 1957, E. Donnall Thomas documented the first human allogeneic hematopoietic stem cell transplantation (allo-HSCT) procedure followed by much of his developmental work on bone marrow and blood-derived HSCT [8–10].

The advent of immunosuppressive drugs led to markedly improved success in preventing graft rejection. In 1972, Cyclosporine A (CsA) was found to be lymphocyte immunosuppressive and was approved in 1980 for use in allo-transplantation [11, 12]. However, CsA and other existing immunosuppressive drugs have deleterious side effects including renal, hepatic, cardiovascular and metabolic dysfunction, opportunistic infections, and secondary malignancies. To improve the quality of life for patients undergoing allo-HSCT, new strategies are evolving to reduce or eliminate the use of immunosuppressive drugs post-HSCT (reviewed in [13]).

**Figure 1:**
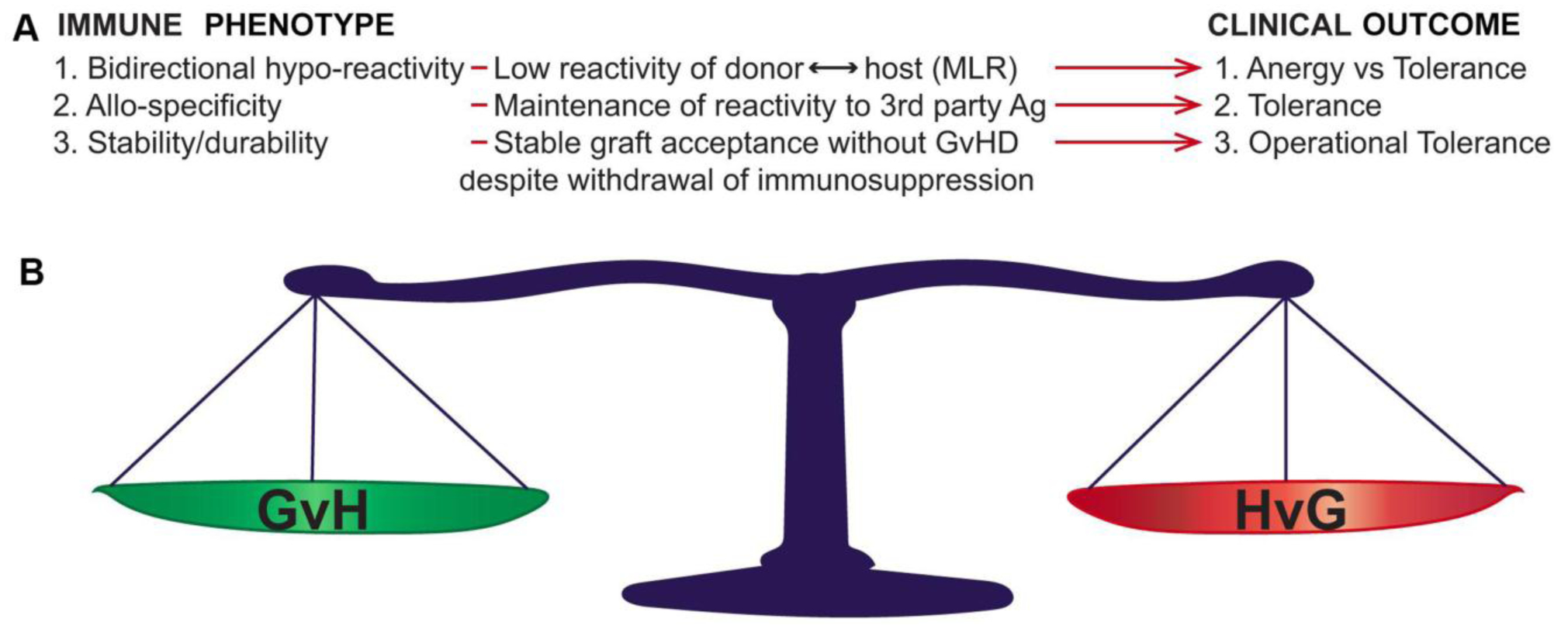
Bidirectional immune features required for operational tolerance (OT) in allo-transplantation. A: Immune correlates and associated clinical outcomes. After OT induction in either HSCT or solid organ transplantation, specific immune phenomena (Immune Phenotype) correlate with specific measurable correlates. The specific clinical outcome associated with each phenotype is defined on the right of the figure (Clinical Outcome). B: OT requires bidirectionality donor recipient immune tolerance. Not only must OT fulfil specific immune criteria in the absence of immune suppression, but this stable long-range balance must be maintained in both the GvH and HvG directions in order to achieve durable graft acceptance without GVHD. MLR, mixed leukocyte reaction; GvH, graft-versus-host reactivity (associated with graft-versus-host disease); HvG, host-versus-graft reactivity (associated with graft rejection).

Non-reactivity between donor and recipient immune systems is defined in specific ways, each with specific clinical correlates (Figure 1A). Lack of responsiveness between donor and recipient without ability to respond to third party antigens (non-specific non-responsiveness) is termed ‘anergy’. Anergy is a form of generalized immune suppression and can clinically associate with lack of robust responses to exogenous or endogenous pathogens. Transplant tolerance is distinct from anergy in that it is donor ←→ recipient alloantigen-specific, with maintenance of reactivity to third party antigens and, importantly, pathogens (reviewed in [7, 14–16]).

## 2. Strategies to Induce Long-Term Immune Tolerance after Allo-transplantation

The distinction between anergy and tolerance (Figure 1) is not merely academic, as multiple translational studies have shown the relevance of antigen-specific hyporeactivity between donor and recipient to the maintenance of a healthy graft without graft-versus-host disease (GVHD), while maintaining host resistance to exogenous pathogens [14, 17–19]. Once antigen-specific tolerance is achieved, the next step is insurance of a stability of this tolerance. This is because maintaining a transplant recipient on lifelong immunosuppression is known to greatly compromise quality of life and increase the risk of transplant-related death from serious infections and drug-associated acute and chronic complications. When (alloantigen-specific) transplant tolerance can be maintained in the absence of drug-induced immunosuppression, this is known as ‘operational tolerance’. To achieve operational tolerance (OT) is the “holy grail” in allo-transplantation and is thus a major focus of preclinical and translational research. Importantly, OT requires bidirectionality (stable maintenance of host-versus-graft and graft-versus-host antigen-specific hyporeactivity) (Figure 1A) in order to be clinically meaningful, as imbalance of either results in graft rejection or GVHD.

## 3. Mixed Chimerism as a Means of Operational Tolerance Induction

One well-studied and translated mechanism for OT induction which does not relate solely on individual immune subsets is the induction of mixed chimerism. This involves rebooting the immune system of the transplant recipient by inducing tolerance to the hematopoietic stem cell (HSC)-derived immune components of the graft donor, and vice versa. After successful OT induction in allo-HSCT, HSCs from the donor and recipient can stably coexist in a patient via induction of host-versus-graft (HvG) and graft-versus-host (GvH) tolerance, also termed “bidirectional immune tolerance” (Figure 1B). This state is characterized by stable co-existence of multilineage hematopoiesis of both recipient and donor, termed ‘stable mixed chimerism’ [20–22]. Additionally, the introduction of an immune system derived from the transplanted HSCs that matches the transplanted organ can result in ongoing thymic deletion of recipient T cell clones capable of rejecting the graft, as well as abrogation of donor T cell clones capable of inducing GVHD. This has successfully achieved solid organ tolerance in human clinical trials. However, such “mixed chimerism tolerogenic protocols” include all of the risks and toxicities associated with HSCT, including short- and long-term toxicity of HSCT conditioning regimens [21, 23, 24]. In this review, we will focus on the roles of innate immune cell subsets of both the donor and recipient immune system in GVHD and donor ←→ recipient transplantation tolerance after HSCT. We will also discuss the potential therapeutic applications of these cells to cure malignant and non-malignant hematological disorders by inducing long-lasting immune tolerance and reducing GVHD.

## 4. Innate Immune Transplant Tolerance

The innate immune system forms the first line of defense against foreign pathogens. Innate immune mechanisms are critical to consider in effecting and maintaining transplantation tolerance across histocompatibility barriers. By definition, these mechanisms are not restricted by major histocompatibility complex (MHC) antigens or, in humans, Human Leukocyte Antigens (HLA). Thus, these components can be harnessed to maintain immune tolerance following HLA-incompatible transplants, as we have shown in murine models of MHC-mismatched HSCT [25–28].

### 4.1 Innate Myeloid Populations

MDSCs, regulatory macrophages, and tolerogenic DCs have all been implicated in allo-HSCT transplant tolerance.

#### 4.1.1 Myeloid-Derived Suppressor Cells (MDSCs)

##### Nomenclature and Phenotype.

MDSCs, expand and recover rapidly after allo-HSCT [29, 30] and can contribute directly to the immunosuppressive tumor microenvironment by dampening T effector cell responses (reviewed in [31, 32]). While this immunosuppressive function may be detrimental in the immune response to cancer, it can be instrumental in achieving transplant tolerance after allo-HSCT [27–30], particularly for non-malignant disorders in which anti-tumor activity is not a consideration. MDSCs constitute a heterogeneous population which can be classified into two broad sub-categories: granulocytic/neutrophilic MDSCs (PMN-MDSCs) and monocytic MDSCs (M-MDSCs) [33–35]. Murine PMN-MDSCs are defined as CD11b^+^Ly6G^+^Ly6C^low^ and M-MDSCs as CD11b^+^Ly6G^neg^Ly6C^high^. In humans, PMN-MDSCs are defined as CD11b^+^CD14^neg^CD15^+^CD66b^+^LOX-1^+^ cells and M-MDSCs are defined as CD11b^+^CD33^+^CD14^+^CD15^neg^CD66b^neg^HLA-DR^low^ cells [35, 36]. Proof of the capacity for T effector cell suppression is obligatory to identify cells as MDSCs. This is because (classical, non-allosuppressive) mature monocytes share many other phenotypic and morphologic features with M-MDSC. The same is true in differentiating neutrophils from PMN-MDSCs; namely, only PMN-MDSCs are known to be allosuppressive [33–35, 37, 38]. In this context, there is some ongoing debate in the field whether MDSCs should be regarded as distinct entities or as monocytes/neutrophils that have been reprogrammed to have immunosuppressive capacity [37–42]. Therefore, better characterization of these cells is needed in order to understand how to phenotypically separate them from other mature myeloid cells.

##### Preclinical Data.

**Figure 2:**
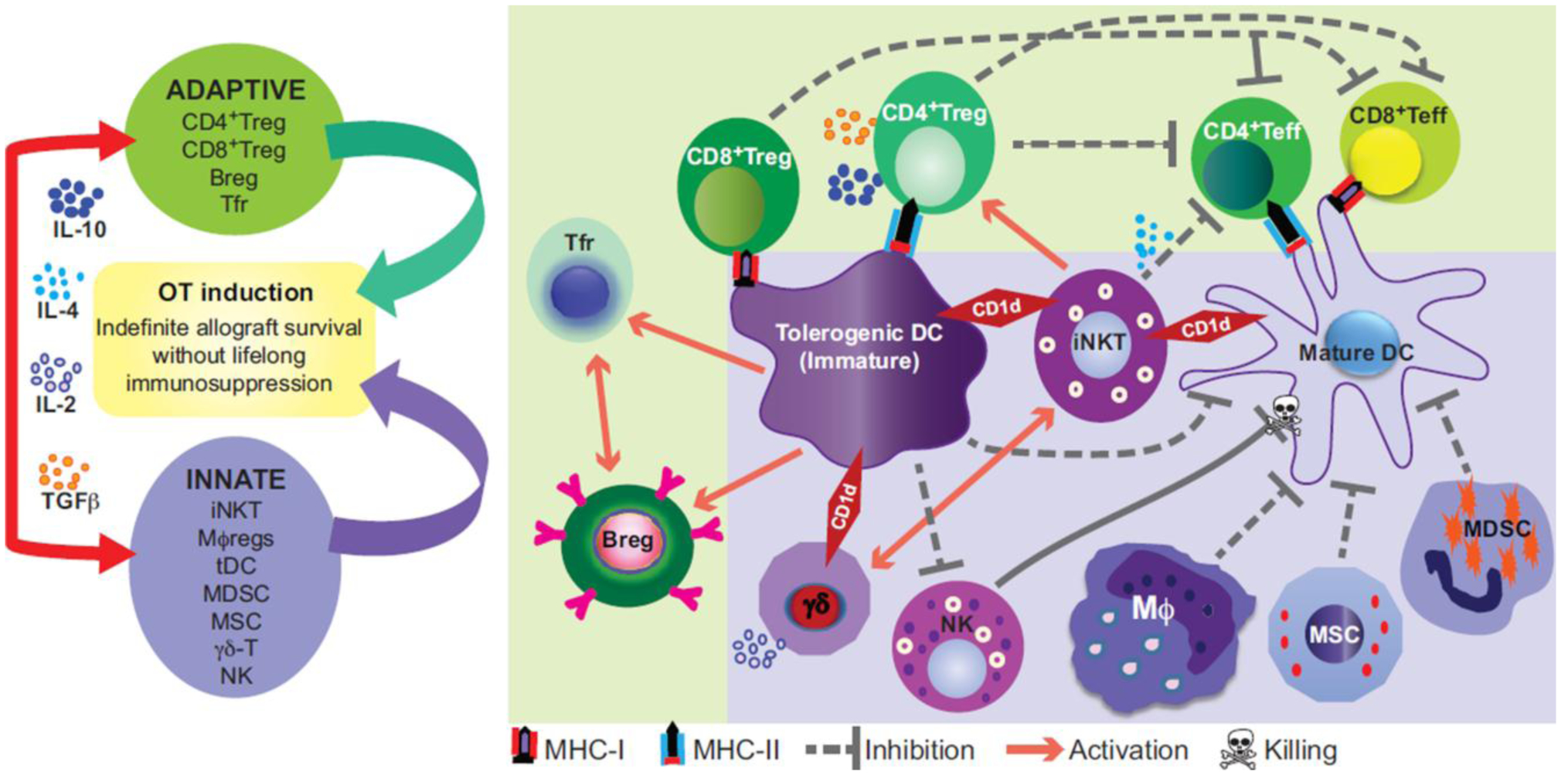
Innate immune networks are a platform for OT induction across histocompatibility barriers. Operational tolerance (OT) is governed through a complex interaction between the innate and adaptive immune systems. The interface between innate *(violet)* and adaptive *(green)* is indicated in the figure and represents the bridging of OT across MHC barriers. iNKT, invariant Natural Killer T cell; MØreg, regulatory macrophage; DC, dendritic cell; MDSC, myeloid- derived suppressor cell; MSC, mesenchymal stromal cell; NK, Natural Killer cell; γδ, gamma-delta T cell; Treg, regulatory T cell; Breg, regulatory B cell; Tfr, regulatory follicular helper T cell; Teff, T effector cell.

In MHC-matched mouse bone marrow transplantation (BMT) studies, donor MDSCs can expand in response to irradiation [43]. Moreover, total MDSC numbers have been correlated to acute GVHD severity in an MHC-mismatched mouse allo-transplant model, and removal of MDSCs aggravates GVHD [44]. In other studies with similar models, donor MDSCs induce allo-HSCT transplantation tolerance by suppressing alloreactive T cells via the enhancement of either arginase-1 or iNOS expression, causing local depletion of L-arginine required for optimal T cell proliferation and survival [45, 46]. Interestingly, Wang *et al.* [44] showed that donor-derived MDSCs from recipients with GVHD were more potent in their inhibition of alloreactive T cell proliferation than donor MDSCs from non-GVHD recipients, suggesting the requirement for an inflammatory immune milieu to optimize MDSC allo-suppressor capacity against GVHD-inducing effector T cells. MacDonald *et al.* [47] demonstrated that donor MDSCs can suppress GVHD by promoting class-II dependent, interleukin (IL)-10 producing T cells. Indeed, infusion of *ex vivo* generated human cord blood MDSCs into a murine allo-HSCT model of chronic GVHD results in regulatory T cell (Treg) expansion thereby attenuating GVHD severity [48]. Tregs are important for controlling alloreactive T cell responses to “self” antigens in the non-transplant setting and for maintaining allograft tolerance in both organ transplant and allo-HSCT [49, 50]. Although detailed mechanisms for MDSC-dependent Treg expansion in allo-HSCT remain undefined, it may be either contact-dependent via programmed death-ligand 1 (PD-L1) as we have shown [27] or mediated via the paracrine production of regulatory cytokines such as transforming growth factor-beta (TGF-β) and IL-10 [45, 51–53]. While the above mentioned work has mainly focused on donor MDSCs in MHC-mismatched allo-HSCT after total body irradiation (TBI) in mice, we have shown that recipient MDSCs and Th2 cytokine signaling are both necessary and required to potently suppress GVHD after nonmyeloablative MHC-mismatched HSCT [27] (Figure 2). We demonstrated in a murine GVHD model system that recipient MDSCs have the ability to convert to regulatory myeloid DCs, which in turn augment donor Treg recovery via a PD-L1 dependent mechanism to induce graft-versus-host immune tolerance [27]. Cumulatively, these data point to MDSC as a potential candidate for cellular immunotherapy, both to prevent and to treat GVHD [44–46, 54]. Toward that end, MDSCs have been expanded *in vivo* with granulocyte-colony stimulating factor (G-CSF) [44], a synthetic fusion of G-CSF and Flt-3 [47], or IL-33 [55]. *In vitro* generation of MDSCs can be achieved by culturing BM cells with granulocyte-macrophage colony stimulating factor (GM-CSF) and G-CSF either with or without addition of IL-13 [45, 46], or a combination of GM-CSF with IL-6 [56]. Zhou *et al.* [54] reported a method to use embryonic stem cells for *in vitro* MDSC differentiation by stimulating these cells with a cocktail of c-kit ligand, vascular endothelial growth factor (VEGF), Flt3 ligand (Flt3L), and thrombopoietin. Notably, relatively high ratios of MDSCs to donor T cells (4:1) appear required for GVHD suppression [45, 54]. Human cord blood MDSC infusion into a xenogenic mouse model has shown a reduction in chronic GVHD of skin, lung, and liver [48].

##### Clinical Data.

Paralleling the data in mice, MDSC numbers have been shown to increase with donor-induced acute GVHD in leukemia patients receiving allo-HSCT [29, 30, 57]. GM-CSF, G-CSF, and IL-6, factors known to promote MDSC expansion, are highly present post-HSCT [57, 58]. CD14^+^HLA-DR^neg/low^IDO^+^ M-MDSCs, as reported by Mougiakakos *et al.*, are not only significantly increased in acute GVHD patients, but can also suppress T cell proliferation *in vitro* [57]. Moreover, inhibition of indoleamine 2,3-dioxygenase (IDO) leads to an almost 3-fold increase in T effector cell proliferation and enhanced interferon-gamma (IFN-γ) release [57]. Aside from T cell suppression and in support of our murine findings [27, 28], human MDSCs have been shown to enhance recovery of both CD4^+^ and CD8^+^ Tregs post allo-HSCT [29, 59]. To date, there are no published trials of human MDSC infusions into patients with active GVHD. MDSCs are a rare population in healthy individuals and therefore difficult to isolate. In order to generate enough M-MDSC numbers for infusion, *ex vivo* expansion procedures are being attempted. These mainly utilize GM-CSF with IL-6 [59], GM-CSF with both IL-6 and VEGF [60], or co-culture with human solid tumor cell lines [60]. Although MDSCs seem promising cellular immunotherapy candidates for inducing and maintaining transplant tolerance, caution is warranted because undifferentiated myeloid subsets are highly adaptable and could theoretically rapidly switch from anti- to pro-inflammatory phenotypes in inflammatory milieus. For example, murine MDSCs are susceptible to inflammasome induction when introduced into a severe inflammatory environment such as that of acute GVHD [61]. Another important therapeutic consideration is that timing of MDSC infusion may be critical to tolerogenesis or to impeding critical anti-viral immunosurveillance [62].

#### 4.1.2 Monocytes and Macrophages

##### Nomenclature and Phenotype.

Macrophages and their (monocyte) precursors rapidly migrate into inflamed tissues in response to injury. Macrophages are the major cell type infiltrating chronically rejected allo-grafts [63] and constitute a phenotypically heterogeneous and remarkably plastic population which can differentiate into a wide spectrum of functional subsets depending upon the tissue microenvironment [63, 64]. In response to different stimuli, infiltrating monocytes preferentially differentiate into “classically activated” or “alternatively activated” macrophages with divergent functions. Classically activated macrophages (M1 macrophages) develop in response to IFN-γ and lipopolysaccharide (LPS), secrete high levels of pro-inflammatory cytokines, and typically promote a Th1 response [65]. These pro-inflammatory M1 macrophages express high levels of arginase-2, inducible nitric oxide synthase (iNOS), tumor necrosis factor-alpha (TNF-α), IL-1, IL-6, and CD86 (reviewed in [66]). In contrast, exposure to Th2-polarizing cytokines leads to the development of alternatively activated M2 macrophages. M2 macrophages are typically regulatory and can be subdivided into M2a, M2b, and M2c subsets, each identifiable by their surface markers and cytokine release profile in response to inflammatory stimuli (reviewed in [66, 67]). All three M2 subsets share the capacity for potent IL-10 secretion. M2a macrophages typically express arginase-1, CD206, CD209, FcεR, and the scavenger receptor CD163 and suppress tissue inflammation [68, 69]. The M2b subset, induced by co-ligation of macrophage FcRs by IgG complexes coupled with toll-like receptor (TLR), CD40 or IL-1R engagement, is immunoregulatory and produces high levels of IL-10, IL-1, IL-6 and TNF-α [66]. M2c tissue repair macrophages are induced with IL-10, express cell-surface SLAM, CD206, and FIZZ1, and produce high amounts of IL-10 and TGF-β [66].

##### Preclinical Data.

Allograft-infiltrating macrophages can cause allograft injury/rejection, tissue remodeling, or graft-site immunoregulation via diverse mechanisms. Thus macrophages can have either protective or detrimental functions in the allo-transplant setting, dependent upon immune phenotypes. For example, M1 macrophages are associated with T cell independent acute allograft rejection. In *acute* GVHD the tissue repair functions of recipient M2 macrophages contribute to resolving inflammation. However, in *chronic* GVHD these same M2 mechanisms can contribute to fibrosis and delayed allograft failure. Specific experimental data follow.

Following myeloablative HSCT, the recipient’s bone marrow (BM) microenvironment is severely damaged due to the cellular stress and tissue injury associated with the pre-conditioning regimen, including adenosine triphosphate (ATP) and Th1 cytokine release, apoptosis of the supporting cells, and microbial antigen leakage from the gut. The latter in turn activates M1 macrophages (reviewed in [65]). M1 macrophages are associated with T cell-independent acute allograft rejection in a murine model of MHC-mismatched allo-HSCT [70–72]. Recipient M1 macrophages can exhibit potent cytotoxicity against the donor BM via the TLR4/toll-interleukin-1 receptor domain-containing adapter inducing interferon-β (TRIF) [71]. TLR4 activation has been identified as a barrier to transplant tolerance (reviewed in [65]). TLR4 recognizes LPS, which can leak from the gut in response to radiation-associated conditioning regimens (reviewed in [73]).

In *acute* GVHD the tissue repair functions of recipient M2 macrophages contribute to resolving inflammation. In contrast to M1 macrophage TLR4 induction of HSCT allograft rejection [71], Imado *et al.* [74] showed a protective role for host macrophages with regards to acute GVHD. In agreement with Imado *et al.* [74], the Merad group has shown that reduction of recipient macrophage numbers results in increased donor T cell expansion, thereby exacerbating acute GVHD after allo-HSCT [75]. Conversely, when recipients were given CSF-1 therapy pre-HSCT (a treatment which expands M2 macrophages), donor T cell expansion was reduced and GVHD morbidity and mortality improved [75]. Recipient macrophages persisting post allo-HSCT are capable of engulfing donor allogeneic T cells directly [75], thus regulating or preventing GVHD. However, in *chronic* GVHD these same M2 macrophage subsets can be detrimental; in mouse models of chronic GVHD, recipient M2 macrophages aggravate the disease by attracting myofibroblasts and increasing TGF-β, inducing massive fibrosis [76, 77]. M2 macrophage polarization can be induced by ER stress, and macrophages subjected to ER stress have been linked to chronic GVHD [78, 79].

##### Clinical Data.

In congruence with the murine preclinical data, an increased ratio of macrophages to total nucleated cells at day 14 post allo-HSCT was associated with delayed engraftment or subsequent graft failure in 32 patients [80]. A genome wide association study (GWAS) in 492 HLA-matched sibling HSCT donor-recipient pairs from Finland and Spain showed that certain single nucleotide polymorphisms (SNPs) in key macrophage functional gene loci such as IL-1R, TNF-α, IL-10, and NFKBIA are associated with *acute* GVHD [81]. In this same study, IL-1B, IL-23R, TLR9, and NOD2 associated with *chronic* GVHD. Interestingly, all these factors are known to play a role in the antimicrobial response of macrophages [82], which follows intestinal damage from the conditioning regimen. In addition, it is known that patients with pre-existing intestinal damage pre-HSCT are more likely to develop acute GVHD after HSCT [83]. Recipient dermal macrophages can be found in GVHD lesions and may persist up to one year post allo-HSCT [84]. Plasma CD163, a macrophage scavenger receptor, has been correlated with a higher cumulative incidence of chronic GVHD in a cohort of 167 patients [85]. Increased CD163 with exposure to the anti-inflammatory cytokine IL-10 may contribute to M2 macrophage recruitment and chronic GVHD pathogenesis [86]. To date, the only attempts to apply regulatory macrophages as a cellular immunotherapy are in renal transplant patients. Hutchinson *et al.* [87] successfully infused 2 patients with donor regulatory macrophages pre-transplantation and maintained graft functionality for 3 years. These regulatory macrophages were generated using M-CSF for 7 days with a 24-hour stimulation with IFN-γ at day 6. This cellular therapy has yet to be tested in allo-HSCT trials. There are some safety concerns with regards to macrophage plasticity after infusion, since these cells change their phenotype based on their specific microenvironment. One could envision mitigating this by timing the infusions pre-HSCT.

#### 4.1.3 Dendritic Cells (DCs)

##### Nomenclature and Phenotype.

As professional antigen presenting cells, DCs are responsible for inducing adaptive immunity essential for priming antigen-specific T cell responses to alloantigens. By regulating these functions, DCs may also promote tolerogenic responses (reviewed in [88, 89]). DCs are heterogeneous, with multiple subsets and distinct functions based on their origin, maturation state, and localization. Mature DCs from the blood include plasmacytoid DCs (pDCs), which are CD11c^lo^MHC-II^l^°, and myeloid DCs (mDCs) characterized as CD11c^hi^MHC-II^hi^. In mice, pDCs can be delineated by their B220 and Siglec-H expression; human pDCs are CD303^+^CD123^+^CD304^+^. Mouse mDCs are recognized as CD24^+^BDCA-1^+^Btla^+^; human mDCs are described as CD11c^+^CD1c^+^BDCA-1^+^ (reviewed in [90]). Designated separately, tolerogenic DCs (tDCs) are a subset of typically immature mDCs with lower MHC-II surface levels, lower expression of co-stimulatory molecules, and a reduced capacity to produce pro-inflammatory cytokines as compared to the mature DCs (reviewed in [89]). In addition to the above mentioned subsets, there are many other types of tissue-resident DCs whose phenotypes and functions have been extensively reviewed elsewhere [90, 91].

##### Preclinical Data.

DC maturation states partially define whether they facilitate or inhibit alloresponses in transplantation, with mature DCs classically defined as potent inducers of alloreactivity and thus of organ rejection and GVHD [92, 93]. Mature DCs present antigen to allogeneic effector T cells, activating them to attack the allograft and inducing graft rejection (reviewed in [94–96]). Most of these rejection studies have been performed in solid organ transplant models (reviewed in [97]). In mice transplanted with MHC-mismatched T cell-depleted BM, surviving recipient cutaneous DCs are sufficient [98] but not required [99] triggers for generation of cutaneous GVHD. Acute GVHD is mostly caused by recipient APCs priming donor T cells in the first few days after allo-HSCT. Like recipient macrophages, recipient DCs can respond to microbial peptides, ATP, and pro-inflammatory cytokines released from the irradiation-damaged gut epithelium and leukocytes. Recipient DC Cathepsin E, an aspartate protease that cleaves bacterial peptides for antigen presentation, regulates DC motility; mice deficient for Cathepsin E are protected from GVHD [100]. Similarly, recipient DC microRNA-155 has been reported as a factor in acute GVHD induction by promoting DC migration towards sites of ATP release [101]. ATP attenuates GVHD by activating the purinergic receptor P2X7R. *Recipient* mice deficient for P2X7R have decreased GVHD and improved survival associated with reduced donor T effector cell expansion and increased CD4^+^Foxp3^+^ Tregs, while *donor* P2X7R deficiency has no effect on GVHD severity or survival [102–104]. Acute GVHD can develop into chronic GVHD when donor mDC MHC-II antigen presentation is impaired, associated with a failure of Treg homeostasis [105]. tDCs can regulate central tolerance by migrating to the thymus, where they may augment negative selection of “self”-/alloreactive thymocytes and the generation of Tregs (reviewed in [106–108]). Unlike mature DCs, tDCs do not express the co-stimulatory molecules necessary to fully activate an alloreactive T effector response. Likely for this reason, maintaining long-range immaturity of DCs has been closely associated with the development of OT. tDCs can maintain OT through multiple mechanisms, including driving naïve T cells to hyporeactivity or inducing T effector cell differentiation into Tregs [109, 110] (Figure 2). Our group defined a critical role for recipient tolerogenic immature myeloid precursors spared by non-myeloablative pre-HSCT conditioning in maintaining MHC-mismatched OT by generating PD-ligand-expressing immature CD8α^neg^ mDCs, which are both Th2 cytokine signaling-dependent and NKT-dependent [28]. These tDCs augment donor-type antigen-specific Treg proliferation through the PD-1/PD-ligand axis [27, 28]. We subsequently defined a means to augment these tDCs *in vivo* by adding alkylators to pre-HSCT conditioning [27].

The role of pDCs in transplant tolerance is less studied. pDC precursor cells (pre-pDCs) have been shown to facilitate allo-HSCT engraftment through Treg induction [111–113]. While data suggest that mature recipient pDCs do not prevent GVHD [114], depletion of pre-pDCs from the donor BM does result in aggravated GVHD [115], supporting a potential tolerogenic role for pDCs in the appropriate milieu. In addition, adoptive transfer of donor pre-pDCs into an MHC-mismatched mouse model of allo-HSCT diminished GVHD [115, 116]. Clearer elucidation of the molecular mechanisms by which tDCs and specific subsets of mDCs and pDCs exert their tolerogenic effects will support their application in OT induction.

##### Clinical Data.

In patients undergoing myeloablative HSCT, donor mDCs and pDCs are already detected in peripheral blood one day after allo-HSCT; within 2 weeks post-HSCT, the majority of circulating DCs are of donor origin [117, 118]. In contrast, human Langerhans cells survive conditioning regimens and persist long after HSCT [119]. After myeloablative pediatric allo-HSCT, patients with acute GVHD had significantly lower numbers of circulating mDCs and pDCs as compared to non-GVHD patients [120, 121]. Chan *et al.* [122] noted that patients with more severe acute GVHD or extensive chronic GVHD had more recipient pDCs than donor pDCs, while patients with low-grade acute GVHD had complete donor pDC reconstitution. Another study found higher peripheral blood *donor* pDC levels in chronic GVHD patients versus non-GVHD patients, while *recipient* pDCs were mostly found in control patients [123]. Also, a high pDC content in the graft was associated with a higher risk of relapse and lower overall survival in patients receiving allo-HSCT [124]. The discrepancies between these studies have been attributed to key differences in conditioning regimens, differences in stem cell source, time of sampling, and pDC identification by flow cytometry, but hey have yet to be meaningfully mechanistically explained.

Infusion of tDCs into a small group of healthy volunteers resulted in an antigen-specific inhibition of T effector cell function [125] and CD8^+^ Treg induction [126]. Though Phase I clinical trials with autologous tDCs in type 1 diabetic [127], rheumatoid arthritis [128], and Crohn’s disease [129] patients show that tDCs are safely tolerated, to date there are no published trials studying tDC immunotherapy after allo-HSCT. In the past 20 years, extensive efforts have been made to generate maturation-resistant tDCs for this purpose, *in vitro*. tDCs are typically differentiated and expanded from PBMCs in the presence of GM-CSF and IL-4 with the addition of 1 or more tolerogenic factors [130, 131] including IL-10, TGF-β, Vitamin D3, dexamethasone, apoptotic cells, corticosteroids, and/or rapamycin [130–140]. Immature tDCs may be a promising alternative to existing regimens for tolerance induction, but require clinical study in the allo-transplant setting.

### 4.2 Innate Lymphoid Populations

Innate lymphoid cells are important as both effector and as regulatory cells, controlling tissue inflammation and immune homeostasis. Since these cells are not MHC restricted in the classic sense, but interact closely with MHC-restricted effector and regulatory T cells, they can be thought of as bridging the innate and adaptive arms of immunity. These lymphocytes develop from a common lymphoid progenitor, but lack the stochastic antigen receptor rearrangements seen in conventional MHC-restricted effector and regulatory T cells. NK cells, NKT cells, and γδ-T cells are all innate lymphoid cells capable of contributing to graft rejection, GVHD, graft-versus-tumor effect, or establishment of OT after allo-HSCT.

#### 4.2.1 Natural Killer (NK) Cells

NK cells can directly kill those leukemic target cells that down-regulate their class I MHC to escape CD8 T cell recognition and killing; they are therefore of great interest in cellular immunotherapy and allo-HSCT.

##### Nomenclature and Phenotype.

Human NK cells are defined as CD3^neg^CD56^+^ and their mouse counterpart is CD3^neg^NK1.1^+^. Human NK cells can be further subdivided into immature lymph node-/tonsil-homed CD56^bright^CD16^neg^ cells and mature peripheral blood-/spleen-homed CD56^dim^CD16^+^. Mature NK cells are predominantly cytotoxic [141]. In mice, 2 mature subsets can be distinguished based on their CD27 expression [142]. NK cell functionality is regulated by several activating and inhibitory receptors which can be either MHC-I specific or non-specific. In mice, MHC-I specific receptors include the Ly49 family and the C-type lectin molecule CD94. Ly49 receptors recognize the MHC-I isoforms H-2D and H-2K. CD94 is covalently associated with NKG2 family members and binds to the MHC class I molecule. In humans, CD94 can bind to non-classic HLA-E molecules on target cells. The human counterparts of the mouse Ly49 family are the killer immunoglobin-like receptors (KIR). This family of receptors can bind to certain groups of the classical class I molecules HLA-C (KIR2DL1–3, KIR2DS1–2, KIR2DS4), HLA-B (KIR3DL2, KIR2DS4), and HLA-A (KIR3DL1) alleles and the non-classical class I HLA-G (KIR2DL4) (reviewed in [143]). Each NK cell expresses a panoply of inhibitory receptors that bind to “self” and/or “non-self” MHC-I molecules. “Licensed” NK cells express inhibitory receptors to self MHC class I, thus allowing them to be inhibited by class I expression on “self” tissue and preventing autoreactivity [144–147].

##### Preclinical Data.

Murine NK cells are highly radioresistant, proliferate under lymphopenic conditions, and thus recover early and rapidly after allo-HSCT [148, 149]. NK cells are potent cytolytic cells which can induce either allograft rejection or OT; the latter by killing allo-responsive activated DCs and T cells (Figure 2). Recipient “licensed” NK cells do not usually recognize donor MHC-I molecules with their KIR and will therefore lyse class I-expressing donor cells, thus inducing graft rejection after allo-HSCT [150].

However, donor NK cell infusions have also been shown to prevent T cell-mediated GVHD, while maintaining or even amplifying the graft-versus-tumor effect [151–155]. GVHD protection was due to the presence of alloreactive “licensed” NK cells capable of killing activated DCs (Figure 2), as supported by the requirement for Ly49 ligand-mismatch from otherwise MHC-matched donors for GVHD control [152, 156] and the fact that Ly49C silencing can induce NK cell alloreactivity [157]. Further emphasizing the importance of NK cell licensing for transplant tolerance, Chow *et al.* [158] showed that the transfer of intact MHC-I antigen from recipient cells to transplanted donor cells confers a “self” identity on these otherwise foreign cells, giving them the ability to evade detection by recipient NK cells. Once complete donor chimerism was established, transplant cells no longer required host MHC-I protein transfer to survive. Alloreactive NK cells can inhibit direct activation of T cells via perforin-mediated killing of allogeneic donor-derived DCs [156, 159–162]. This does not occur with unprimed T cells [163], supporting that the NK cell effect acts through DC ablation by NK cells. Conversely, non-licensed inflammatory NK cells can contribute to GVHD development by pro-inflammatory Th1 cytokine production [164–166].

##### Clinical Data.

In patients undergoing allo-HSCT, NK cells are the first donor-derived lymphocytes to reconstitute [167, 168]. Donor NK cells show a robust proliferation immediately after HSCT [169] and multiple studies suggest that higher NK cell numbers reduce GVHD incidence [170–177]. The dominant NK phenotype in the first weeks post-HSCT is CD56^bright^CD16^neg^, while healthy individuals have only 10% CD56^bright^CD16^neg^ and 90% CD56^dim^CD16^++^ NK cells [178–180]. In most patients, the relative NK cell expansion normalizes 12 months post-HSCT [168]. Levels of recipient versus donor immune subset chimerism may play a role in allo-HSCT. Breuer *et al.* [181] identified 3 risk groups for leukemia relapse in children after reduced intensity or myeloablative HSCT, based on recipient chimerism (RC): 1) a high-risk group with both T cell and NK cell RCs above 90%; 2) an intermediate-risk group with a T cell RC above 50% and NK cell RC below 90%; and 3) a low-risk group with a T cell RC below 50%. Though the data defined that low donor-type NK cell chimerism correlates with relapse, the association with GVHD was not reported.

In acute GVHD, reconstitution of donor NK cells is often delayed, and acute GVHD patients 2 months post-HSCT show lower numbers of CD56^bright^ NK cells [168]. A prolonged increase in the number of CD56^int^ NK cells has been observed in allo-HSCT patients with chronic GVHD [168]. As in murine pre-clinical studies, the presence of alloreactivity is required for human NK cell-mediated GVHD protection [182–189]. Most of these studies have focused on donor and/or recipient KIR receptor genotypes and haplotypes [182–185]. Lack of activating KIRs on donor NK cells (expected to be required for DC killing by NK cells) can increase GVHD risk [184]. When donor and recipient KIR genotypes are matched, the risk of chronic GVHD is diminished [183, 185]. Next to matching donor and recipient KIR haplotypes, matching of donor KIR genotype with recipient HLA genotype appears to influence GVHD risk [186]. Donor→recipient combinations described worthy of avoidance in allo-HSCT are KIR2DL2→HLA-C2, KIR2DS2→HLA-C1, and KIR2DS1→HLA-C2 [186]. Recently, Hu *et al.* [190] noted a decrease in NKG2A^+^ NK cells in patients with GVHD as compared to those without GVHD after allo-HSCT. *In vitro*, NKG2A^+^ NK cells were able to suppress T cell IFN-γ production. Moreover, NKG2A^+^ NK cells were able to induce Tregs upon naive T cell co-culture [190]. In support of this, higher proportions of IFN-γ producing NK cells post-HSCT are associated with increased cumulative incidence of GVHD [191]. To summarize, it is important for OT induction that donor NK cells are “licensed” and that their inhibitory KIR are mismatched with the HLA ligands of recipients. Therefore, donor NK cell phenotype and recipient KIR and/or HLA status are important to consider when applying NK cells in allo-HSCT, not only for anti-tumor effects but also for tolerance induction [192]. Human *ex vivo* expanded NK cells have just completed phase I/II clinical trials. Whereas most of these have not shown successful GVHD reduction [193–196]. Lee and colleagues [197] did demonstrate GVHD reduction in patients given NK cell infusions. While others infused the NK cells after HSCT, Lee *et al.* did so prior to HSCT. Pre-clinical studies support that timing for NK cell infusion may be important for GVHD prevention [151, 154]. Conversely, Shah *et al.* [198] reported aggravated acute GVHD upon NK cell infusion. Unlike the other trials, patients in this study received pre-activated NK cells. Production of pro-inflammatory cytokines from these pre-activated NK cells may in part explain why GVHD was worsened in this study. Though NK cells are established key effectors in anti-tumor immunotherapy, a better understanding of mechanisms employed by NK cells to direct immunotolerogenesis versus alloreactivity is needed to advance the application of NK cells in transplant tolerance induction.

#### 4.2.2 Natural Killer T (NKT) Cells

##### Nomenclature and Phenotype.

NKT cells (reviewed in [199]) share morphological and functional characteristics with both T cells and NK cells. Human NKT cells are mainly identified as CD3^+^TCRαβ^+^CD161^+^CD56^+^. Cytokines secreted by NKT cells can have powerful effects on αβ-T cell Th1/Th2 differentiation. NKT cells’ ability to rapidly respond without first having to differentiate into T effector cells places them at the front lines of defense against pathogens. NKT cells recognize antigen in the form of glycolipids presented by the MHC-I-like molecule CD1d on APCs; therefore, like NK cells, NKT cells are not restricted to MHC-I/II driven responses. NKT cells can be divided into 2 main subsets: Type I NKT cells, also known as invariant NKT (iNKT), and the less well understood type II NKT cells. iNKT cells express an invariant TCRα-chain (Vα14-Jα18 in mice and Vα24-Jα18 in humans) and can be further subdivided into CD4^+^CD8^neg^, CD4^neg^CD8^neg^ (termed “double negative” in mice and humans), and a rarer subset which is CD4^neg^CD8^+^ [199].

##### Preclinical Data.

Murine iNKT cells are particularly resistant to radiation-induced apoptosis due to their relatively high levels of anti-apoptotic factors including bcl-2 [200]. iNKT cells can rapidly secrete large amounts of cytokines including IL-4 upon activation [25, 199]. We have demonstrated that preservation of iNKT cells in BM of recipient mice receiving MHC class I and class II-mismatched allo-HSCT was required for maintenance of bidirectional donor ←→ recipient immune tolerance and that iNKT cells allowed the maintenance of antitumor activity in the setting of HSCT for recipient-derived lymphoma [25, 26]. We and others have shown, first in murine models and then in clinical HSCT, that this iNKT axis can be manipulated for transplant tolerance induction after allo-HSCT using reduced toxicity conditioning [25, 26, 201–203]. Others have since recapitulated that treatment of recipient mice by a variety of methods that enhance iNKT cell numbers or Th2 polarization can reduce GVHD morbidity and mortality [204–208]. Dominant innate immune mechanisms we have reported and/or others have recapitulated for iNKT cell-induced tolerance (Figure 2) include IL-4-dependent augmentation of PD-1 ligand-expressing recipient myeloid suppressor cells that induce contact-dependent expansion of donor thymically-derived Treg cells [25–27], costimulatory (CD40-CD40L) blockade of iNKT cells [207], direct inhibition of CD8^+^ T effector cells [25, 209], and iNKT-associated generation of other subsets of tolerogenic CD8^+^ DCs [210]. In addition, there are other mechanisms not yet defined by which iNKT-derived Th2 cytokines may induce OT [200–202, 209]. Adoptive transfers of iNKT cells with and without prior *in vitro* expansion have been successfully shown by us and other groups to reduce acute GVHD without impeding engraftment or anti-tumor activity [25, 26, 211–215].

##### Clinical Data.

As important support for the relevance of iNKT cells to human immune tolerance, multiple groups in large-scale clinical studies have shown a strong association of patient overall survival and protection from GVHD on either iNKT cell graft content before HSCT [216] or early quantitative iNKT immune recovery after HSCT [217, 218]. Peripheral blood stem cell CD4^−^ iNKT cell dose was shown as the only graft parameter to predict significant acute GVHD, supposedly by selective suppression of T cell proliferation and IFN-γ secretion [216]. Just as in the pre-clinical setting, the same reduced toxicity pre-HSCT conditioning in patients increased IL-4 production by CD4^+^ T cells, elevated iNKT cell numbers, and reduced cumulative incidence of acute GVHD [19, 219–221]. In our own data [222] and two separate recent publications [223, 224], *ex vivo* expanded human iNKT cells were able to regulate T cell activation and proliferation while having direct *in vitro* and *in vivo* cytotoxicity against specific malignancies [225]. Thus cumulative current data indicate that iNKT cell-based immunotherapy is promising for reducing GVHD and allowing successful tolerance induction. Further investigation of tissue-specific iNKT interaction with other regulatory components of the immune system will likely pave the way for novel clinical protocols using human iNKT cells for tolerance induction after either HSCT or organ transplantation.

#### 4.2.3 Gamma-Delta T (γδ-T) Cells

##### Nomenclature and Phenotype.

γδ-T cells share many activating and inhibitory receptors with NK cells, and activation of these cells similarly depends on the net balance of signals from both types of receptors [226]. Like iNKT cells, γδ-T cells are CD3^+^ yet their TCR (in this case, TCRγδ) does not bind with antigen presented on conventional MHC molecules (reviewed in [227]). Instead, these cells recognize a broad range of ligands including phospholipids and small unprocessed peptides (reviewed in [228]). They can attack target cells directly through cytotoxic activity or indirectly through the activation of other immune cell subsets. γδ-T cell functional responses are induced upon the recognition of stress antigens, promoting cytokine production and regulating pathogen clearance [229]. In humans there are two major subsets of γδ-T cells identified by their Vδ chains. Vδ1-T cells are predominant in the thymus and peripheral tissues. Vδ2-T cells constitute the majority of circulating γδ-T cells [229]. Human γδ-T cells maintain a Vγ9 chain and mainly recognize phosphorylated non-peptide molecules that are metabolic intermediates of the isoprenoid biosynthesis pathway [229, 230].

##### Preclinical Data.

γδ-T cells are detected as early as 1 month post allo-HSCT in murine models using graft TCRαβ depletion [231]. The role of γδ-T cells in allo-tolerance is not entirely clear [229–235], particularly in mouse models. γδ-T cells have been reported to both reduce and increase GVHD depending upon the experimental model [231–235]. Drobyski *et al.* [232, 236] showed that γδ-T cells do not induce GVHD. However, Blazar *et al.* [233] showed that the infusion of donor γδ-T cells did induce lethal GVHD in mice and Maeda and colleagues [234] observed similar effects of host γδ-T cells. In another study by Ellison *et al.* [235], elimination of these cells from donors significantly reduced GVHD and post-HSCT mortality.

##### Clinical Data.

Partially mismatched or haploidentical allo-HSCT causes marked increases in the γδ-T cell pool and results in reduced GVHD in allo-HSCT for both non-malignant [231] and malignant disorders [237–239] (Figure 2), while allowing increased anti-tumor activity in allo-HSCT for malignancies [237–239]. Development of large-scale clinical methods for enriching, isolating, expanding, and therapeutically manipulating γδ-T cells holds promise to ameliorate GVHD while maintaining anti-infectious immunity and enhancing long-term survival after allo-HSCT [237–239].

### 4.3 Mesenchymal Stromal Cells (MSCs)

#### 

##### Nomenclature and Phenotype.

MSCs are a fibroblast-like, heterogeneous, non-hematopoietic, pluripotent progenitor cell population with self-renewal potential (reviewed in [240, 241]). MSCs are plastic-adherent and can be expanded *in vitro*. In humans, MSCs are characterized by the expression of CD73, CD90, and CD105 but absence of CD14, CD34, CD45 and HLA-DR. MSCs directly and indirectly suppress T cell reactivity (reviewed in [240, 241]).

##### Preclinical Data.

Infusion of allogeneic MSCs can induce immune tolerance to skin allografts [242]. MSCs have since been shown to rapidly migrate to sites of inflammation, including transplanted organs. MSCs exert immunomodulatory effects on a wide array of immune cells already known to play direct roles in transplantation tolerance, including T cells, B cells, NK cells, monocytes/macrophages, and DCs [242–245]. It is important to functionally distinguish MSCs (a stromal cell population derived from BM microenvironment) from MDSCs (immature myeloid hematopoietic cells of non-stromal origin) [243]. MSCs can arrest activated T cells in the G0/G1 phase and decrease their production of IFN-γ and IL-2 [243]. When exposed to inflammatory stimuli, as in the recently transplanted organ, MSCs display favorable immunomodulatory potential. This, combined with their unique migratory properties, make them strong theoretical candidates for site-specific control of allograft inflammation [242–245]. Cho *et al.* [245] demonstrated that soluble factors in medium conditioned by human tonsil MSCs attenuates acute GVHD in a mouse model. Overall, MSC infusion in multiple transplant models results in a skew of the balance between regulatory and effector T cells towards a tolerogenic profile [243, 244].

##### Clinical Data.

Data support a role for MSCs in facilitating HSC engraftment and bidirectional tolerance, particularly in the setting of pediatric allo-HSCT [246–248]. In particular, donor-derived MSC co-transplantation following haploidentical allo-HSCT can facilitate engraftment with low rates of GVHD for severe aplastic anemia in children [248]. Ball *et al.* [249] showed that co-transplantation of *ex vivo* expanded MSCs may reduce the risk of graft failure in haploidentical HSCT. To interrogate the clinical utility of MSCs in reducing GVHD, a meta-analysis of randomized controlled trials (RCTs) was conducted [250]. In chronic GVHD patients with hematological malignancies undergoing allo-HSCT, umbilical cord-derived, high-dose, and late-infused MSCs prevented chronic GVHD (Figure 2), while bone marrow-derived, low-dose MSCs or MSCs co-infused with the graft appeared to be ineffective [250]. Though limited by the usual caveats of a meta-analysis, the study suggested that high-dose umbilical cord blood-derived MSC infusion should be explored in the RCT setting as a prophylactic strategy to reduce chronic GVHD after allo-HSCT. Three active clinical trials for HLA-matched and HLA-mismatched allo-HSCT in European, Israeli, and Korean consortia applying cord blood, bone marrow, or adipose tissue-derived MSCs to prevent or treat acute GVHD have yet to report their findings on safety and efficacy.

## 5. Future Directions

Medical and scientific advances since the first tissue transplants have greatly expanded the applications of both allo-HSCT to cure hematopoietic and immune disorders and organ transplantation to prolong survival after organ failure. Nonetheless, relatively few significant strides have been made in the past 30–40 years toward long-range allograft survival and successful wean from pharmacological immunosuppression. Significant advances have recently been made in allo-HSCT by using reduced toxicity regimens that enhance recipient ←→ donor immune interactions and immune regulatory cell function. Other approaches remain limited by a requirement for long-term immunosuppression, associated drug toxicity, and infectious and oncologic sequelae of chronic non-specific immune suppression. Our increased understanding of the regulatory cells and molecular pathways involved opens the opportunity for their exploitation to prevent and/or treat GVHD and graft rejection after allo-HSCT and, by extension, to prevent or treat organ allograft rejection. The theoretical advantages of the innate cellular immune regulatory approach are: 1) OT induction that obviates a need for ongoing immunosuppression and 2) alloantigen specificity, which reduces the risk of compromising immune responses to exogenous pathogens. To date, a number of key candidates have been identified in rigorous pre-clinical studies which appear to be translating well to human investigations, including iNKT cells, myeloid suppressor populations (MDSCs), and stromal cells (MSCs). Despite initial technical challenges relating to large-scale expansion, these candidates are now undergoing clinical trials. Results from these and other studies over the next decade will likely greatly advance our understanding of the optimal regimens, the most appropriate cell sources (donor, host, third party), and any potential targeting of these cells required to improve long-range operational tolerance induction.
